# Size‐assortative choice and mate availability influences hybridization between red wolves (*Canis rufus*) and coyotes (*Canis latrans*)

**DOI:** 10.1002/ece3.3950

**Published:** 2018-03-23

**Authors:** Joseph W. Hinton, John L. Gittleman, Frank T. van Manen, Michael J. Chamberlain

**Affiliations:** ^1^ Warnell School of Forestry and Natural Resources University of Georgia Athens GA USA; ^2^ Odum School of Ecology University of Georgia Athens GA USA; ^3^ U.S. Geological Survey Northern Rocky Mountain Science Center Interagency Grizzly Bear Study Team Bozeman MT USA

**Keywords:** assortative mating, body size, *Canis latrans*, *Canis rufus*, coyote, hybridization, monogamous breeding, red wolf, reproductive barriers, space use

## Abstract

Anthropogenic hybridization of historically isolated taxa has become a primary conservation challenge for many imperiled species. Indeed, hybridization between red wolves (*Canis rufus*) and coyotes (*Canis latrans*) poses a significant challenge to red wolf recovery. We considered seven hypotheses to assess factors influencing hybridization between red wolves and coyotes via pair‐bonding between the two species. Because long‐term monogamy and defense of all‐purpose territories are core characteristics of both species, mate choice has long‐term consequences. Therefore, red wolves may choose similar‐sized mates to acquire partners that behave similarly to themselves in the use of space and diet. We observed multiple factors influencing breeding pair formation by red wolves and found that most wolves paired with similar‐sized conspecifics and wolves that formed congeneric pairs with nonwolves (coyotes and hybrids) were mostly female wolves, the smaller of the two sexes. Additionally, we observed that lower red wolf abundance relative to nonwolves and the absence of helpers increased the probability that wolves consorted with nonwolves. However, successful pairings between red wolves and nonwolves were associated with wolves that maintained small home ranges. Behaviors associated with territoriality are energetically demanding and behaviors (e.g., aggressive interactions, foraging, and space use) involved in maintaining territories are influenced by body size. Consequently, we propose the hypothesis that size disparities between consorting red wolves and coyotes influence positive assortative mating and may represent a reproductive barrier between the two species. We offer that it may be possible to maintain wild populations of red wolves in the presence of coyotes if management strategies increase red wolf abundance on the landscape by mitigating key threats, such as human‐caused mortality and hybridization with coyotes. Increasing red wolf abundance would likely restore selection pressures that increase mean body and home‐range sizes of red wolves and decrease hybridization rates via reduced occurrence of congeneric pairs.

## 1 INTRODUCTION

Under Mayr's (1942) biological species concept, the origin of species involves reproductive isolation and evidence still favors the view that new species usually arise as byproducts of evolution in geographically isolated populations (Coyne & Orr, 2004; Hey, Fitch, & Ayala, 2005; Pfennig & Pfennig, 2010; Schluter, 2001). Global environmental change caused by human activity has eliminated many geographic barriers that prevented secondary contact between closely related taxa that arose through allopatric speciation. Secondary contact and reproductive interactions facilitate hybridization among formerly allopatric populations with divergent evolutionary lineages. Although some studies have presented hybridization as a positive force that provides beneficial adaptive genetic variation from one species to another (Abbott et al., 2013; Brennan et al., 2014; Stebbins, 1959; vonHoldt, Brzeski, Wilcove, & Rutledge, 2017), others have attributed hybridization and introgression as a threat to imperiled populations and species (Genovart, 2009; Ellstrand et al., 2010; Rhymer & Simberloff, 1996; Todesco et al., 2016). Indeed, the literature pertaining to reproductive barriers and speciation is voluminous, but its broader integration into conservation and management has been underappreciated (Allendorf, Leary, Spruell, & Wenburg, 2001; Rhymer & Simberloff, 1996; Seehausen, Takimoto, Roy, & Jokela, 2008; vonHoldt, Brzeski et al., 2017).

Natural hybridization is observed more frequently in certain taxonomic groups, as 25% of plant and 10% of animal species surveyed in studies are known to hybridize (Mallet, 2005), and hybridization tends to concentrate in specific geographic regions (e.g., hybrid zones; Barton & Hewitt, 1985; Benson, Patterson, & Wheeldon, 2012; Swenson & Howard, 2005). For example, despite birds having greater speciation rates and achieving greater species diversity than mammals, they evolve complete hybrid inviability at slower rates than mammals (Fitzpatrick, 2004; Wilson, Maxon, & Sarich, 1974). Several stable and well‐studied avian hybrid zones occur across significant areas of the Great Plains of the United States, where ranges of 14 pairs of geographically separated species overlap (Curry, 2005; Dixon, 1989; Mettler & Spellman, 2009). Hybridization among mammal species in the Great Plains is relatively rare (Shurtliff, 2013), but hybridizing species of several mammalian genera, such as *Canis* (Kyle et al., 2006; Nowak, 2002; Rutledge, Garroway, Loveless, & Patterson, 2010), *Geomys* (Genoways, Hamilton, Bell, Chambers, & Bradley, 2008; Heaney & Timm, 1985), and *Odocoileus* (Cathey, Bickham, & Patton, 1998; Stubblefield, Warren, & Murphy, 1986), have historically occurred. Regardless of taxonomy, populations of congeners are more likely to interact reproductively during secondary contact if they are recently diverged sister taxa (Coyne & Orr, 2004), similar in some ecological, morphological, and behavioral traits (Crossman, Taylor, & Barrett‐Lennard, 2016; Montanari, van Herwerden, Pratchett, Hobbs, & Fugedi, 2011), and exhibit a poor ability to discriminate between species (Gill & Murray, 1972; Myers & Frankino, 2012).

In particular, reproductive isolation of coyotes (*Canis latrans*), eastern wolves (*Canis lycaon*), and red wolves (*Canis rufus*) is incomplete, in which gene flow occurs between them via hybridization and introgression, and likely has done so for much of their evolutionary history where their ranges overlapped (Brzeski, DeBiasse, Rabon, Chamberlain, & Taylor, 2016; Kyle et al., 2006; Rutledge, Devillard, Boone, Hohenlohe, & White, 2015; Rutledge, Garroway et al., 2010). However, agricultural conversion of natural habitats and predator control programs that extirpated wolf populations facilitated coyote range expansion into the historic ranges of eastern wolves and red wolves during the 20th century (McCarley, 1962; Nowak, 2002; Rutledge, White, Row, & Patterson, 2012; Stronen et al., 2012). Research suggests that limited population growth of wolves caused by excessive anthropogenic mortality was the primary cause facilitating hybridization between the two eastern North American wolf species and coyotes (Benson, Patterson, & Mahoney, 2014; Bohling & Waits, 2015; Hinton, Brzeski, Rabon, & Chamberlain, 2017; Rutledge, White et al., 2012). As a result, research and management priorities for wolf conservation in eastern North America focused on understanding the extent to which reproductively compatible but ecologically different *Canis* taxa may coexist with minimal levels of gene flow (Benson et al., 2014; Gese et al., 2015; Rutledge, Wilson, Klütsch, Patterson, & White, 2012).

Endemic to the eastern United States, red wolves share a common ancestor with coyotes and differentiated from them in allopatry during the Pleistocene but began interbreeding with coyotes in the southeastern United States during the early 20th century, when remnant wolf populations began interacting with expanding coyote populations (Chambers, Fain, Fazio, & Amaral, 2012; Nowak, 2002, 2003; Wilson et al., 2000). By 1980, the red wolf was extirpated from the wild but, via a captive breeding program, reintroduced into eastern North Carolina beginning in 1987 (Hinton, Chamberlain, & Rabon, 2013; United States Fish and Wildlife Service [USFWS], 1989). Meanwhile, coyotes rapidly colonized the red wolf's historic range and currently co‐occur with the small reintroduced wolf population in eastern North Carolina (Gese et al., 2015; Hinton & Chamberlain, 2014). Because hybridization with coyotes is a primary challenge to red wolf recovery, the USFWS Red Wolf Recovery Program (Recovery Program) implemented the Red Wolf Adaptive Management Plan to minimize hybridization and prevent coyote introgression via sterilization of coyotes paired with wolves (Gese & Terletzky, 2015; Gese et al., 2015).

Fundamentally, hybridization results from mate choice by individual red wolves. Previous assessments warned that female red wolves pairing with coyotes (Kelly, Miller, & Seal, 1999) and a lack of reproductive barriers between wolves and coyotes (Fredrickson & Hedrick, 2006) would be problematic for red wolf recovery. Studies following those assessments reported that anthropogenic factors, specifically gunshot mortalities during the breeding season, facilitated hybridization by disrupting red wolf breeding pairs with a greater proportion of female wolves than males breeding with coyotes (Bohling & Waits, 2015; Hinton, Brzeski et al., 2017). However, coyote introgression into the wild red wolf population remained <4% because the Recovery Program's use of coyotes and hybrids as sterile placeholders provided an artificial reproductive barrier (Gese & Terletzky, 2015). Although sterile placeholders limited introgression, studies of hybridization (Bohling & Waits, 2015; Bohling et al., 2016) and breeding pair dynamics (Hinton, Brzeski et al., 2017) observed nonrandom mating in the reintroduced population, suggesting that assortative mating was also playing a role in limiting the extent of hybridization (Bohling et al., 2016).

Factors influencing assortative mating in *Canis* taxa are likely multifaceted with a diversity of behavioral and ecological correlates that may influence hybridization (Benson & Patterson, 2013; Bohling et al., 2016; Hinton et al., 2013; Hinton, Ashley et al., 2017; Rutledge, Garroway et al., 2010; Rutledge, White et al., 2012). Essentially, hybridization results when individual red wolves and coyotes consort to form congeneric breeding pairs that defend territories and produce hybrid litters (Hinton et al., 2013; Hinton, Brzeski et al., 2017; Hinton, Ashley et al., 2017). Long‐term monogamy, defense of all‐purpose territories, and group living that involves bi‐parental care of offspring are core characteristics of *Canis* (Bekoff, Diamond, & Mitton, 1981; Geffen et al., 1996; Gittleman, 1989; Kleiman, 2011) and behaviors associated with consorting, mate selection, and mate fidelity may serve as behavioral reproductive barriers that prevent hybridization among sympatric *Canis* taxa. For example, studies routinely report that gray wolves (*Canis lupus*) and coyotes are reproductively isolated in the wild (García‐Moreno, Matocq, Roy, Geffen, & Wayne, 1996; Hohenlohe et al., 2017; Kyle et al., 2006; Pilgrim, Boyd, & Forbes, 1998; Rutledge, Wilson et al., 2012; Wheeldon, Patterson, & White, 2010), although it has been suggested that the two species do hybridize (vonHoldt et al., 2011, 2016; vonHoldt, Cahill et al., 2017). Gray wolf and coyote interactions are well documented throughout North America and, despite routinely interacting ecologically as sympatric species (Arjo, Pletscher, & Ream, 2002; Atwood & Gese, 2010; Switalski, 2003), amicable consorting behavior between them is extremely rare (Hohenlohe et al., 2017; Thiel, 2006). To our knowledge, congeneric pairings between gray wolves and coyotes have not been confirmed in field studies. However, congeneric pairings between red wolves and coyotes are well documented (Gese & Terletzky, 2015; Hinton, Brzeski et al., 2017; Hinton, Ashley et al., 2017), implying that red wolves and coyotes are capable of sharing space and food resources to overcome limited mating opportunities.

Red wolf and coyote breeding pairs exhibit constrained movements over the landscape, as site fidelity is expressed by their consistent use and territorial defense of specific localities via passive (i.e., scent marking) and aggressive (i.e., physical conflict) behaviors to exclude conspecifics (Benson & Patterson, 2013; Gese & Terletzky, 2015; Hinton, van Manen, & Chamberlain, 2015; Hinton et al., 2016). These space use patterns comprise behaviors that reflect how both species use their environment in response to internal and external pressures. For example, space use is positively correlated with carnivore body mass, where larger carnivores require larger territories than smaller carnivores to fulfill greater energetic requirements (Gompper & Gittleman, 1991; McNab, 1963). Indeed, Hinton, van Manen et al. (2015) reported that coyote home ranges in eastern North Carolina ranged between 13 and 47 km^2^ and suggested that coyote body size constrained the area they could effectively exploit and defend as territories. Furthermore, Ward (2017) assessed space use of 147 coyotes radio‐marked with Global Positioning System (GPS) collars in Alabama, Georgia, and South Carolina and reported that 80% of resident coyotes maintained home ranges below 20 km^2^. The larger body size of red wolves allows them to, on average, maintain larger territories than coyotes, but some red wolves maintain similar home‐range sizes as coyotes (Hinton et al., 2016). Because cooperation and coordination between breeding pairs for both species is crucial for efficient foraging, parental care of offspring, and territory defense, Hinton, Ashley et al. (2017) hypothesized that when individual red wolves require home ranges larger than consorting coyotes can maintain, asymmetric exploitation of space between larger wolves and smaller coyotes may prevent congeneric pairings. If this is true, then assortative mating observed between red wolves and coyotes may result from size‐based choice, as asymmetry in partner sizes may make it too costly to strive for the best available options required by the larger or smaller mates (Schuett, Tregenza, & Dall, 2010; Taborsky, Guyer, & Taborsky, 2009; Taborsky & Taborsky, 1999).

Currently, it is unknown whether innate preferences or environmental conditions are responsible for reproductive barriers observed in *Canis* taxa, but both conditions likely play an important role facilitating hybridization. It is widely acknowledged that human‐mediated mortality of wolves disrupts the social structures of wolf packs and reduces their abundance on the landscape (Benson et al., 2014; Borg, Brainerd, Meier, & Prugh, 2015; Hinton, White, Rabon, & Chamberlain, 2017; Hinton, Brzeski et al., 2017 *;* Milleret et al., 2017; Rutledge, Patterson et al., 2010). Because gray wolves and coyotes do not exhibit consorting behaviors that lead to congeneric pairings, even when wolf densities are low, there is no interaction between human‐caused mortality and hybridization between gray wolves and coyotes in western North America (Hohenlohe et al., 2017; Wheeldon et al., 2010). Conversely, red wolves and coyotes can form congeneric pairs likely because red wolves and coyotes are sibling species that have recently diverged (Hohenlohe et al., 2017; Kyle et al., 2006; Rutledge et al., 2015; Wilson et al., 2000) and have not evolved strong discriminatory behaviors that facilitate complete reproductive isolation. However, behavioral traits that promote assortative mating and prevent congeneric pairings likely restrict gene flow between red wolves and coyotes (Bohling et al., 2016; Fredrickson & Hedrick, 2006). It is currently unknown how morphological and behavioral differences between red wolves and coyotes influence consorting behaviors, but mate choice for these species has long‐term consequences and breeding pairs should coordinate behaviors efficiently to defend territories and improve offspring survival. Therefore, similarity in body size and space use behaviors are likely two important innate traits influencing assortative mating between red wolves and coyotes, as these traits likely provide information on the behavioral consistency and quality of mates that they attempt to pair‐bond with. Now that geographic barriers (e.g., pre‐Columbian landscapes) have been eliminated and the only wild population of red wolves co‐occurs with coyotes, it is essential to identify behaviors influencing potential reproductive barriers between wolves and coyotes. If reproductive barriers do exist, they may represent one of the only opportunities to maintain a wild population of red wolves in the presence of coyotes. In this study, we used a detailed data set on red wolf mate selection spanning 20 years to investigate factors influencing wolf mating patterns and hybridization with coyotes.

## 2 MATERIAL AND METHODS

### 2.1 Study area

**Figure 1:**
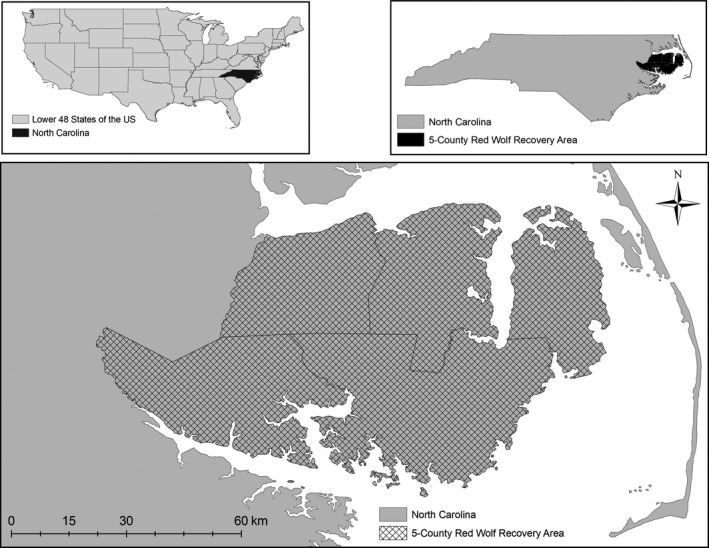
The Red Wolf Recovery Area on the Albemarle Peninsula of northeastern North Carolina

Comprising approximately 6,000 km^2^ of federal, state, and private lands, the Red Wolf Recovery Area was located on the Albemarle Peninsula in northeastern North Carolina (Figure 1). The landscape consisted of a row‐crop agricultural‐bottomland forest matrix in which agricultural crops (i.e., corn, cotton, soybean, winter wheat) and managed pine (*Pinus* spp.) comprised approximately 30% and 15% of vegetative cover, respectively. Other prominent vegetative cover on the Albemarle Peninsula included coastal bottomland forests and pocosin (35%), herbaceous wetlands and saltwater marshes (5%), and other minor vegetative communities (10%). Further details of the study area can be found in Hinton, Ashley et al. (2017).

### 2.2 Capture and monitoring

Since 1987, Recovery Program biologists annually trapped red wolves to fit individuals with mortality‐sensitive very‐high‐frequency (VHF; Teleonics, Mesa, AZ) and Global Positioning System (GPS; Lotek 4400S, Newmarket, Ontario, Canada) radio collars and regularly monitored radio‐marked wolves until individuals died or radio collars failed (Hinton, White et al., 2017). By 1992, coyotes began colonizing the Recovery Area and the first hybridization event occurred during 1993 (Gese et al., 2015). Subsequently, coyotes were trapped, fitted with radio collars, and monitored by the Recovery Program (Gese & Terletzky, 2015; Hinton, Brzeski et al., 2017). Therefore, red wolves and coyote monitored for this study occurred during 1992–2012 when consorting behavior between the two species were observed. Methods to capture, handle, and process red wolves and coyotes were in cooperation and concordance with the USFWS, approved by the Louisiana State University Agricultural Institutional Animal Care and Use Committee (Protocol Number AE2009‐19), and met guidelines recommended by the American Society of Mammalogists (Sikes & Gannon, 2011).

During October through May of each year, red wolves and coyotes were captured using padded foot‐hold traps (Victor no. 3 Softcatch, Woodstream Corporation, Lititz, PA). Ages, species identity, and parentage of captured red wolves were known if individuals were carrying a subcutaneous passive integrated transponder (PIT) tags inserted into the animal during annual surveys of red wolf dens (Gese et al., 2015; Hinton, White et al., 2017). Ages of red wolves without PIT tags and coyotes were estimated by tooth wear (Gipson, Ballard, Nowak, & Mech, 2000), and a blood sample was taken to determine parentage and species identity. Coyotes were taken to a local veterinary clinic for surgical sterilization (Gese & Terletzky, 2015). This procedure reduced hybridization and introgression and allowed the Recovery Program to use sterile coyotes as placeholders until those coyotes were displaced by red wolves or were removed for management reasons (Gese & Terletzky, 2015). Once red wolves and coyotes were fully processed, individuals were fitted with radio collars, released, and then monitored by the Recovery Program during weekly telemetry flights. Weekly monitoring efforts via aircraft allowed the Recovery Program to identify and monitor territories of radio‐marked red wolves and coyotes on the landscape.

Breeding pairs were identified as radio‐collared individuals of breeding age (≥2 years old) that were temporally and spatially associated with one another and were defending a territory for ≥6 months (Hinton, Brzeski et al., 2017). Only three types of *Canis* breeding pairs were routinely monitored by the Recovery Program: red wolves (2 red wolves), coyotes (2 coyotes), and congeneric (red wolves with coyotes or hybrids). Biologists confirmed breeding pair status of red wolves during spring den visits (March–May) by locating dens and daybeds of females to verify the presence of litters of known, radio‐collared breeding pairs (Beck, Lucash, & Stoskopf, 2009). Congeneric pairs and coyote pairs were confirmed through field monitoring and occasionally by den visits if coyotes and hybrids had not been captured and sterilized.

### 2.3 Data analyses

Many *Canis* breeding pairs disbanded under natural and anthropogenic causes, in which widowed red wolves and coyotes replaced mates by either maintaining their territories and pair‐bonding with transients or becoming transients themselves to seek out new mates and territories (Hinton, van Manen et al., 2015; Hinton et al., 2016; Hinton, Brzeski et al., 2017). Consequently, many red wolves in our study had multiple mates during their lifetime. Therefore, we assessed breeding history for red wolves monitored by the Recovery Program during 1992–2012 and classified pairings into two categories: conspecific (red wolves that paired with red wolves) and congeneric (red wolves that paired with coyotes or hybrids).

Similar to previous studies (Bohling & Waits, 2015; Hinton, Brzeski et al., 2017), we used qualitative descriptions of specific events experienced by each red wolf when they formed conspecific and congeneric breeding pairs to assess whether anthropogenic mortality (e.g., shooting deaths) facilitated congeneric pairings. We simplified this category and assigned red wolves to one of two categories: those that were widowed or were in packs that disbanded because of gunshot mortality and those that were not. Because some red wolf breeders had established packs with juveniles (Hinton & Chamberlain, 2010; Sparkman, Adams, Steury, Waits, & Murray, 2010; Sparkman et al., 2011), we also classified wolves in pairing events as either having helpers or not when acquiring a new mate to assess if pack structure influenced congeneric pairings. To examine the influence of breeder experience on acquiring conspecific and congeneric mates, we classified red wolves in pairing events as first‐time breeders or experienced breeders (Bohling & Waits, 2015). Because some red wolves were represented in multiple pairing events, there were interdependencies in our data. We accounted for such interdependencies in our univariate analyses by including random intercepts for individual red wolves in generalized linear mixed models (GLMM) in R (R Core Team, 2014; Bates, Maechler, Bolker, & Walker, 2015) that compared the frequency of gunshot mortalities, helpers, and first‐time breeders between conspecific and congeneric pairings. We then used the likelihood ratio test as a means to attain *p*‐values by comparing the likelihood of the model with a factor to the intercept‐only model.

The *Canis* population in our study area consisted of a continuum of canids with body masses ranging between 7 and 39 kg that red wolves could form breeding pairs with (Hinton & Chamberlain, 2014). To assess the influence of body size on congeneric pairings, we used body traits of red wolves, coyotes, and hybrids that were recorded for individuals, while they were processed and fitted with radio collars (Hinton & Chamberlain, 2014). Body traits measured included body mass, body length (anterior tip of the nose pad to the tail base), tail length (tip of the fleshy part of the tail to the tail base), hind foot length (hock to the tip of the digital pads), shoulder height (tip of the scapula to tip of the digital pads), length of head (edge of the premaxillary to the most posterior point of the occipital bone), width of head (widest points across the zygomata), and ear length (edge of the external auditory canal to the tip of the ear). We used a principal component analysis (PCA; JMP software; SAS Institute) to create a single measurement of overall body size. Based on Brzeski, Rabon, Chamberlain, Waits, and Taylor (2014), we assumed the PCA segregated variation due to body size by linearly combining such variation into the first principal component (PC1). We used the restricted maximum likelihood (REML) method to create a completed data set to perform the PCA and address missing values within our morphometrical data set (Paul & Peng, 2009). We only included individuals ≥10 months of age in the PCA, as these canids approached their potential adult sizes and achieved the minimum physical size to safely wear radio collars. We then created a measurement of mate similarity between red wolves and their mates by dividing PC1 values of breeding pairs. For our univariate analyses, and to account for interdependencies caused by red wolves involved in multiple breeding events, we included random intercepts for individual wolves in GLMM analyses that compared sex and similarity values of red wolves involved in conspecific and congeneric pairings. We again used the likelihood ratio test to attain *p*‐values by comparing the likelihood of the model with a factor to the intercept‐only model.

To estimate space use patterns, we calculated home ranges of red wolves and coyotes that had ≥30 telemetry locations during the period they were paired with a mate using Geospatial Modeling Environment (GME; Beyer, 2014) and ArcMap 10.3 (Environmental Systems Research Institute 2014). We created annual home ranges for individual red wolves and coyotes in breeding pairs by calculating 95% fixed kernel density estimates using the h‐plugin smoothing parameter within GME (Seaman & Powell, 1996; Worton, 1989). Because some red wolves were represented in multiple pairing events, our univariate analyses included random intercepts for individual red wolves in GLMM analyses comparing home‐range sizes of red wolves involved in conspecific and congeneric pairings. We used the likelihood ratio test as a means to attain *p*‐values by comparing the likelihood of the model with a factor to the intercept‐only model.

We used trapping data to calculate annual ratios of red wolves to nonwolves (coyotes and hybrids) during 1992–2012 to estimate an index of red wolf abundance (Hinton, Brzeski et al., 2017). Annual trapping efforts were not standardized temporally or spatially, because Recovery Program biologists also coordinated with private fur trappers to capture as many *Canis* taxa as possible within the 5‐county Recovery Area. Nevertheless, trapping efforts supporting the large‐scale, long‐term monitoring efforts conducted across the Recovery Area provided a reasonable proxy for relative abundances of *Canis* species (Hinton, Brzeski et al., 2017; Stephens, Pettorelli, Barlow, Whittingham, & Cadotte, 2015). We used linear regression to assess whether annual red wolf to nonwolf ratios declined through time.

We used pairings as a binary response variable (1 = conspecific, 0 = congeneric) in a GLMM with a logit link in R (Bates et al., 2015) to investigate factors that influenced mate selection by red wolves. These factors included sex of red wolves, body size ratio between mates, wolf home‐range size, annual wolf to nonwolf ratios, anthropogenic‐caused breakups of breeding pairs, the presence of helpers, and previous breeding experience of wolves. We included random intercepts for red wolves to account for individual variation. Prior to modeling, we rescaled values for all continuous variables by subtracting their mean and dividing by two standard deviations (Gelman, 2008) and conducted correlation analysis to ensure that independent variables were not highly correlated (*r *<* *.7).

To develop an ecologically meaningful a priori set of models, we used seven general hypotheses (Table 1) to test factors that may influence congeneric pairings between red wolves and coyotes. First, we included a binary variable for sex (1 = female, 0 = male) because previous studies (Bohling & Waits, 2015; Hinton, Brzeski et al., 2017) observed more female red wolves paired with coyotes than males. Second, we included a body size ratio between breeding pairs derived from our PCA as a measurement of mate similarity because body size was the primary morphologic trait distinguishing red wolves from nonwolves (Hinton & Chamberlain, 2014) and hypothesized to influence congeneric pairings (Hinton, Rabon et al., 2015; Hinton, Ashley et al., 2017). Third, we included home‐range sizes of individual red wolves for each breeding pair event because we hypothesized that space use behaviors were likely an important behavior influencing assortative mating (Hinton et al., 2016; Hinton, Ashley et al., 2017). Fourth, we included annual red wolf to nonwolf ratios because we hypothesized that the availability of wolf mates influenced congeneric pairings (Benson et al., 2012; Bohling & Waits, 2015; Hinton, Brzeski et al., 2017; Rutledge, White et al., 2012). Fifth, we included a binary variable for anthropogenic‐caused breakups of breeding pairs (1 = pairing occurred after the loss of a mate to gunshot mortality, 0 = pairing did not occur after the loss of a mate to gunshot mortality) because anthropogenic mortality can facilitate *Canis* hybridization (Benson et al., 2014; Bohling & Waits, 2015; Hinton, Brzeski et al., 2017; Rutledge, Wilson et al., 2012). Sixth, we included a binary variable for pack structure (1 = presence of helpers, 0 = no helpers) because pack structure has been identified as an important trait preventing hybridization (Bohling & Waits, 2015; Rutledge, Patterson et al., 2010). Finally, we included a binary variable for breeder experience (1 = first‐time breeder, 0 = experienced breeder) because Bohling and Waits (2015) reported that first‐time female breeders were responsible for a significant proportion of hybridization events. Red wolves in pairing events that lacked body measurements or home‐range data were censored from our GLMM analysis. We then selected variables for our multivariate GLMM analysis using the univariate tests of each hypothesis, considering only variables with significant tests (Bursac, Gauss, Williams, & Hosmer, 2008). We based this on our likelihood tests and a *p*‐value cutoff of .25, as more traditional levels (e.g., 0.05) can fail to identify important variables (Bursac et al., 2008). We then used Akaike's information criterion adjusted for small sample sizes (AIC_c_) and used ΔAIC_c_ to select which models best supported factors influencing congeneric pairings between red wolves and nonwolves (Burnham & Anderson, 2002).

**Table 1:** A selection of ecological factors as potential predictors of congeneric pairings between red wolves and coyotes

Factors	Link to breeding pair formation	Sources
Red wolf to mate body size ratio	Congeneric pairings more likely between coyotes and wolves when they are similar in body size	Hinton, Rabon et al. (2015), Hinton et al. (2016), Hinton, Ashley et al. (2017)
Home‐range size	Congeneric pairings more likely between coyotes and wolves when wolves maintain small home ranges (e.g., ≤50 km^2^)	Hinton, Rabon et al. (2015), Hinton et al. (2016), Hinton, Ashley et al. (2017)
Red wolf to nonwolf ratio	Congeneric pairings more likely when coyotes outnumber wolves	Benson et al. (2012), Rutledge, White et al. (2012), Bohling and Waits (2015), Hinton, Brzeski et al. (2017)
Presence of helpers	Congeneric pairings more likely between solitary wolves and coyotes	Rutledge, Patterson et al. (2010), Rutledge, White et al. (2012), Bohling and Waits (2015)
Gunshot mortalities	Congeneric pairings more likely following disruption of packs by gunshots	Rutledge, Patterson et al. (2010), Rutledge, White et al. (2012), Benson et al. (2014), Bohling and Waits (2015), Hinton, Brzeski et al. (2017)
Sex	Congeneric pairings more likely between female wolves and male coyotes	Bohling and Waits (2015), Hinton, Brzeski et al. (2017)
First mating event	Congeneric pairings more likely between coyotes and young, inexperienced wolves	Bohling and Waits (2015)

## 3 RESULTS

**Figure 2:**
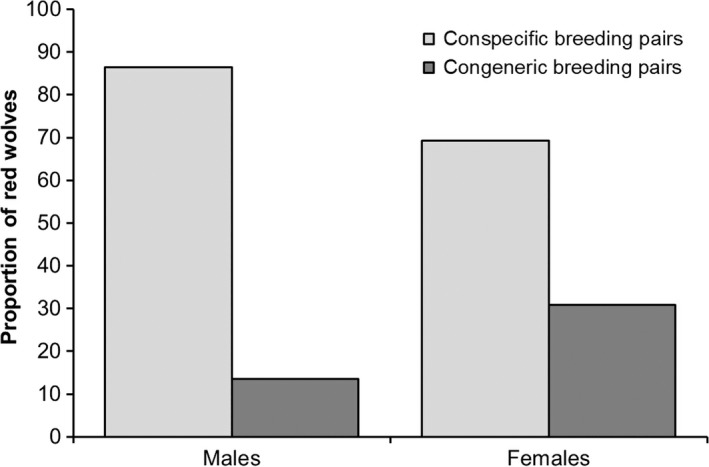
Proportion of red wolves involved in conspecific and congeneric pairings in northeastern North Carolina, 1992–2012

During 1992–2012, we identified 131 pairing events involving 96 red wolves (51 males, 45 females) that successfully formed breeding pairs with wolves and nonwolves (coyotes and hybrids). Conspecific pairings comprised 79% (104 of 131), whereas 21% were congeneric pairings. Approximately 86% (57 of 66) of pairings involving male red wolves were conspecific, whereas 72% (47 of 65) of pairings involving females were conspecific, as females were more likely to pair with nonwolves than males ($\chi_{1}^{2}$ = 5.69, *p *=* *.017; Figure 2). Although the frequency of helpers was slightly greater for conspecific pairings than congeneric ($\chi_{1}^{2}$ = 2.82, *p *=* *.093), we observed no difference in the frequency of first‐time breeders ($\chi_{1}^{2}$ = 0.89, *p *=* *.347) and gunshot mortalities ($\chi_{1}^{2}$ = 0.02, *p *=* *.901) between conspecific and congeneric pairings. Finally, annual red wolf to nonwolf ratios declined from 1992 through 2012 (*r* ^2^ = .64, *p *<* *.001).

Our morphometric data set consisted of measurements recorded from 462 red wolves, 252 coyotes, and 161 hybrids during 1987–2012. The first PC explained 60% of the cumulative variation in our data (Table 2). The eigenvector of PC1 had similar loadings that were all positive, indicating that PC1 primarily accounted for variation in body size. Mean PC1 scores differed among red wolves, coyotes, and hybrids for females (*F*_2,420_ = 323.11, *p *<* *.001) and males (*F*_2,453_ = 383.32, *p *<* *.001), as hybrids were intermediate in size to red wolves and coyotes for both sexes (see Hinton & Chamberlain, 2014). Male ($\chi_{1}^{2}$ = 19.93, *p *<* *.001) and female ($\chi_{1}^{2}$ = 61.72, *p *<* *.001) red wolves in conspecific pairs were more similar in body size to their mates than those in congeneric pairs. Mean body size ratios between male red wolves and their mates in conspecific and congeneric pairings were 1.20 ± 0.18 and 1.72 ± 0.49, respectively, in which male wolves were typically larger than their female mates. However, mean body size ratios for female red wolves and their mates in conspecific and congeneric pairings were 0.92 ± 0.13 and 1.40 ± 0.30, respectively, in which female wolves were typically the smaller mate when paired with wolves but the larger mate when paired with nonwolves.

**Table 2:** Eigenvalues, share of total variance along with eigenvectors, and factor loadings of body measurements of red wolves in northeastern North Carolina, 1992–2012. Significant loadings shown in bold

Body measurements	Principal component 1		Principal component 2	
	Eigenvector	Loading	Eigenvector	Loading
Body mass	0.40	**0.87**	−0.13	−0.12
Ear length	0.32	**0.69**	0.25	0.24
Tail length	0.23	**0.52**	0.74	**0.72**
Body length	0.35	**0.75**	−0.18	−0.18
Hind foot length	0.39	**0.85**	0.26	0.25
Shoulder height	0.38	**0.84**	−0.20	−0.19
Head length	0.39	**0.84**	−0.03	−0.03
Head width	0.34	**0.74**	−0.46	−**0.45**
Eigenvalue	5.76		0.93	
% of total variance	59.46		11.57	

**Figure 3:**
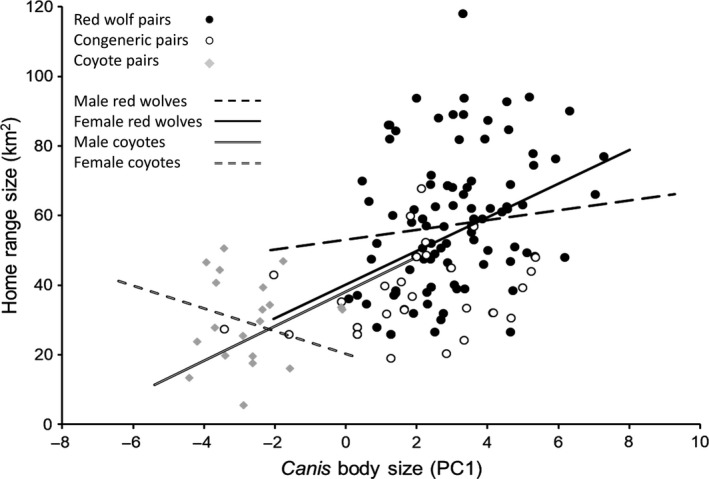
Correlation between home‐range size and body size of male (*r* ^2^ = .047, *p *=* *.075) and female (*r* ^2^ = .081, *p *=* *.091) red wolves and male (*r* ^2^ = .142, *p *=* *.077) and female (*r* ^2^ = −.080, *p *=* *.583) coyotes in breeding pairs, northeastern North Carolina, 1992–2012. Correlation for all *Canis* was *r* ^2^ = .268 (*p *<* *.001)

**Figure 4:**
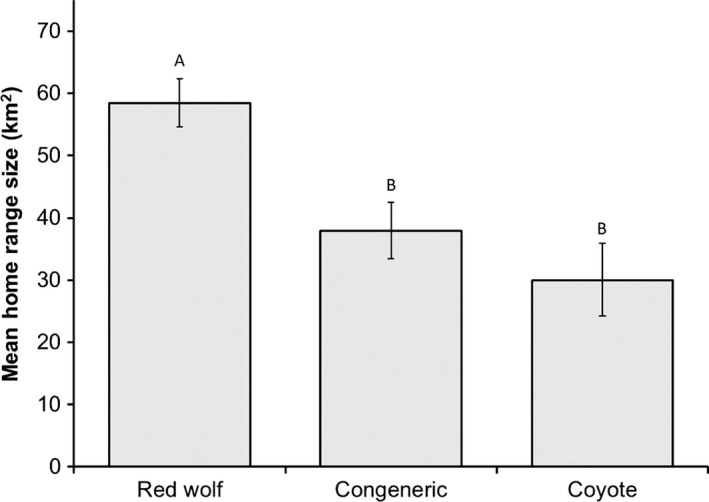
Mean home‐range sizes of red wolf, congeneric, and coyote breeding pairs in northeastern North Carolina, 1992–2012. The 95% confidence intervals are represented by the error bars. Letters above the bars represent statistical differences among breeding pair categories (P < 0.05, Tukey’s test)

Of 131 pairing events, mean size and standard deviation of red wolf home ranges were 54.2 km^2^ ± 19.4 and ranged between 19.0 and 118.0 km^2^. Of 23 coyotes, mean size and standard deviation of home ranges were 30.0 km^2^ ± 11.7 and ranged between 5.5 and 50.6 km^2^. Mean home‐range size of red wolves was greater than coyotes ($\chi_{1}^{2}$ = 30.83, *p *<* *.001). When pooled, the body size of red wolves and coyotes involved in pairings was positively correlated with home‐range sizes ($\chi_{1}^{2}$ = 43.91, *p *<* *.001; Figure 3). Body size was not correlated with the size of home ranges for female coyotes ($\chi_{1}^{2}$ = 0.33, *p *=* *.583), whereas there was a weak positive correlation for males ($\chi_{1}^{2}$ = 3.10, *p *=* *.077). Body size was weakly correlated with size of home ranges for female ($\chi_{1}^{2}$ = 2.83, *p *=* *.091) and male ($\chi_{1}^{2}$ = 3.17, *p *=* *.075) red wolves. Red wolves in conspecific pairs had larger home‐range sizes than wolves in congeneric pairs, whereas home‐range sizes of wolves in congeneric pairs were similar to coyote home‐range sizes ($\chi_{1}^{2}$ = 49.53, *p *<* *.001; Figure 4).

When developing our models, we excluded the factors of breeder experience and gunshot mortality because we observed no significant effect of these factors on mating patterns in our univariate analyses. Therefore, we included five factors (sex, body size ratios between mates, red wolf home‐range size, annual red wolf to nonwolf ratio, and the presence of helpers) in our multivariate analysis. The global model best explained factors influencing assortative mating in red wolves (Table 3). The two strongest parameters in our model were body size ratios of mates and sex of red wolves, as decreasing body size ratios between red wolves and their mates was strongly associated with conspecific pairs and male wolves occurred proportionately more often in conspecific pairs than did females (Table 4). The strong effect of body size similarity and sex in our model suggests that red wolves prefer mates of similar size and that male wolves may have stronger preferences for larger mates than do females. Furthermore, home‐range size of red wolves was positively correlated with conspecific pairs and suggests that wolves with large home‐range sizes were involved in conspecific pairs more often than wolves with small home ranges (Table 4). The annual wolf to nonwolf ratios was positively associated with conspecific pairs and the presence of helpers exerted a weak positive correlation with conspecific pairs, suggesting that red wolf abundance and pack structure increases the probability that wolves will acquire conspecific mates (Table 4).

**Table 3:** Generalized linear mixed models for predicting probability of congeneric breeding corresponding to different hypotheses of factors associated with breeding pair formation by red wolves in northeastern North Carolina, 1992–2012. Shown are differences among Akaike's information criteria for small sample sizes (ΔAIC_c_)

Model structure	*k*	Deviance	ΔAIC_c_	AIC_c_ω
SR^a^ + HR^b^ + W:C^c^ + Helpers^d^ + Females^e^	7	39.50	0.0	0.70
SR + HR + W:C + Females	6	45.20	2.88	0.17
SR + HR + Helpers + Females	6	46.80	3.86	0.10
SR + HR + Females	5	51.50	7.02	0.02
SR + W:C + Helpers + Females	6	52.40	8.05	0.01

a Red wolf to mate size ratio.

b Red wolf home‐range size.

c Red wolf to nonwolf ratio.

d Number of helpers in pack.

e Red wolf sex.

**Table 4:** Results from generalized linear mixed models for the global model for predicting probability of congeneric breeding corresponding to different hypotheses of factors associated with breeding pair formation by red wolves in northeastern North Carolina, 1992–2012. Shown are β coefficients, standard error (*SE*), 95% confidence intervals (CI), *z*‐scores, and *p*‐values

Model variables	β	*SE*	95% CI	*z*	*p*
Intercept	4.213	0.956	2.654, 6.966	4.407	<.001
Red wolf to mate size ratio	−2.856	0.657	−4.752, −1.759	−4.439	<.001
Home‐range size	2.085	0.858	0.718, 4.200	2.430	.014
Red wolf to nonwolf ratio	1.272	0.601	0.227, 2.715	2.119	.034
Presence of helpers	3.007	1.918	0.129, 7.486	1.568	.117
Females	−3.165	1.067	−5.851, −1.303	−2.965	.003

## 4 DISCUSSION

Recent studies on *Canis* hybridization in eastern North America have suggested that prey selection (Rutledge, Garroway et al., 2010) and territorial aggression (Benson & Patterson, 2013) may play a role in reducing hybridization, but stressed that excessive human‐caused mortality of wolves ultimately facilitated conditions for hybridization between wolves and coyotes in eastern North America. Additionally, Bohling et al. (2016) found mating to be nonrandom and assortative between red wolves and coyotes in eastern North Carolina, in which most hybridization events were correlated with excessive anthropogenic mortality and often involved young female wolves (Bohling & Waits, 2015). The results of our study largely are, but not completely, confirmatory of these and other previous studies in that suggested behavioral (space use), demographic (availability of mates), social (presence of helpers), and sex‐biased (females) factors influence mating patterns of red wolves. Additionally, our results suggest that red wolves likely seek mates of matching body size, indicating that assortative mating between wolves and coyotes may be size‐related. This is not surprising as behaviors associated with space use, diet, and interspecific interactions of carnivores are constrained by their body size and energetic demands (Carbone, Teacher, & Rowcliffe, 2007; Donadio & Buskirk, 2006; Gittleman & Harvey, 1982) and, for long‐term monogamous breeders, choosing a partner assortatively from a behavioral perspective could be advantageous if similar individuals are capable of coordinating their behaviors better than nonassortative pairs (Schuett et al., 2010). However, local environmental variables that influence mating patterns, such as population density, are largely influenced by anthropogenic factors. In other words, human‐caused mortality reduces red wolf abundance on the landscape and increases the probability of wolves interacting with coyotes, but mate similarity and varying space use behaviors of wolves influences which individuals are capable of forming congeneric pairs with coyotes. However, we found no association between gunshot mortality and congeneric pairings, despite previous studies that suggested shooting deaths are a primary driver of red wolf survival and population size (Hinton, White et al., 2017) and are positively correlated with hybridization events (Bohling & Waits, 2015) and congeneric pairings (Hinton, Brzeski et al., 2017).

The link between behavioral traits and mating patterns in hybridizing *Canis* taxa remains relatively unexplored, but our results provide some novel insights and suggest that assortative mating in *Canis* likely involves multiple causes. For instance, the red wolf to non‐wolf ratio had a positive association with conspecific pairings, consistent with the mate availability hypothesis, where the spatial distribution of potential mating partners influences the probability of encountering conspecifics (Crespi, 1989; Pal, Erlandsson, & Sköld, 2006; Rowe & Arnqvist, 1996). This is not surprising given that wolf density has been the primary commonality among studies of *Canis* hybridization, with low wolf densities caused by anthropogenic mortality facilitating outbreeding with coyotes by eastern wolves (Benson et al., 2012, 2014; Rutledge, Patterson et al., 2010; Rutledge, White et al., 2012) and red wolves (Bohling & Waits, 2015; Hinton, Brzeski et al., 2017). Additionally, low red wolf to nonwolf ratios likely influenced the positive association of female wolves and lack of helpers with congeneric pairings. Previous studies of gray wolves and eastern wolves reported female‐biased subordinated breeding and male‐biased dispersal to packs where dispersers filled vacant male breeding positions (Jędrzejewski et al., 2005; Rutledge, Patterson et al., 2010; vonHoldt et al., 2008). Similarly, the sex‐bias in conspecific pairings suggests that male and female breeders may employ different strategies to compensate for the loss of mates, in which widowed females exhibit stronger fidelity to territories than widowed males and, consequently, acquire new mates from the transient population. Because coyotes greatly outnumber red wolves, female wolves likely interact more often with transient coyotes than transient wolves after the loss of a mate. When red wolf densities are low, transient coyotes are more likely to interact with solitary red wolves, in which successful pairings may depend primarily on the adequacy of coyotes to deal with environmental factors, such as habitat and prey availability. However, as red wolf densities increase (e.g., greater wolf to nonwolf ratios), coyotes are more likely to encounter widowed red wolves cohabiting territories with offspring (helpers) from previous mates and are then subjected to social selection that involves winning interactions with other pack members while contesting to be a breeder (West‐Eberhard, 1983). Therefore, greater red wolf to nonwolf ratios increases the probability that wolves interact more often with wolves than coyotes and increase kin‐based social structures that discourage amicable consorting with coyotes.

Home‐range size of red wolves was an important variable in our models, as extensive space use behaviors of wolves was positively correlated with conspecific breeding. This is consistent with the mating constraint hypothesis that suggests various costs of mating, such as physical or energetic barriers, create difficulties during courtship, copulation, or mate guarding (Arnqvist, Rowe, Krupa, & Sih, 1996; Crespi, 1989; Harari, Handler, & Landolt, 1999). In particular, red wolves generally maintained larger home ranges than coyotes, but home‐range sizes of wolves and coyotes overlapped in the 25–50 km^2^ range (Figure 4). Approximately 87% of congeneric pairs had home ranges within 25–50 km^2^, whereas the remaining home ranges were between 52 and 68 km^2^. Because foraging and territorial defense are energetically demanding activities, it is likely that significant differences in potential spatial (e.g., territory size) and dietary (e.g., predation on white‐tailed deer [*Odocoileus virginianus*]) requirements between consorting red wolves and coyotes discourages congeneric pairings. Ultimately, when red wolves and coyotes are capable of consorting, a primary factor that leads to successful pairings appears to be establishing territories below 50 km^2^, a range of home‐range sizes that coyotes can adequately maintain and defend.

Our analyses indicated that reduced body size ratios between red wolves and their mates were the most important variable in our models, as 79% of observed wolf pairings were conspecific despite that wolves were generally outnumbered by coyotes. Assortative mating based on similarity in size is one of the most prevalent mating patterns in the animal kingdom, and it is known to act as a premating reproductive barrier between distinct species and divergent populations (Coyne & Orr, 2004; Galipaud, Bollache, & Dechaume‐Moncharmont, 2013; Jiang, Bolnick, & Kirkpatrick, 2013). Therefore, it is reasonable to assume that effects of body size were manifested in *Canis* space use patterns (Figure 3), in which red wolves with smaller home ranges were more likely to be congeneric breeders than those with larger home ranges. Space use was positively correlated with *Canis* body mass in eastern North Carolina and, because coyotes are smaller than red wolves, the upper limit to the areas coyotes could effectively exploit and defend as territories was below the average home‐range size of wolves. The low proportion of red wolves ≥27.5 kg observed in congeneric pairings may indicate an important threshold, as most wolves above that threshold did not form breeding pairs with coyotes and hybrids. Although this trend was largely driven by male red wolves, the larger of the two sexes, dissimilarity in body size between congeneric pairs and their small home ranges suggests a potential cost when congeneric pairs attempt to achieve territory sizes large enough to accommodate the wolf's greater energetic requirements but small enough for coyotes to defend. As a species with long‐term monogamy, acquiring mates and territories are critical events for red wolves and likely require extensive mate assessment before new pairs are formed. Female red wolves likely choose males that behave similarly to themselves in use of space and diet, as these behaviors are consistent and may allow females to identify which males can provide high levels of territorial defense and parental care. However, because female red wolves are closer in body size to coyotes and hybrids than males (Hinton & Chamberlain, 2014), they are likely more capable of reconciling the costs of having smaller coyote or hybrid mates and can likely compensate and adjust their space use and foraging behaviors accordingly to successfully breed with dissimilar mates.

It is important to understand what circumstances facilitate hybridization and how it affects the persistence of imperiled species and, where possible and desired, to mitigate irreversible consequences such as genetic swamping and loss of phenotypic uniqueness. It is not surprising that our results highlight mate similarity in body size and space use behaviors as important factors preventing congeneric pairings, because territorial behavior is a fundamental life history strategy for *Canis* taxa. Nearly all of our study area was occupied by territories of red wolves and coyotes and, because vacant territories were commonly occupied by transients, there was intense competition for space. Territorial turnover for red wolves and coyotes typically occurs after the death of resident breeders, as surviving residents are receptive to acquiring new mates from the transient population (Hinton, van Manen et al., 2015; Hinton et al., 2016; Hinton, Brzeski et al., 2017). Similar to gray wolves (Milleret et al., 2017), it is rare for healthy red wolves and coyotes to divorce their mates to acquire new ones, and therefore, transients of both species typically encroach into territories experiencing mortality and replace lost resident breeders. For widowed red wolves, the predominant risk is the loss of the territory and the loss of a partner may be detrimental if a widowed wolf is not able to defend the territory against intruders. Therefore, widowed residents may seek more contacts with, and be less aggressive toward, potential partners because quick repairing is crucial for widows to keep their territories (Hinton, van Manen et al., 2015; Hinton et al., 2016; Hinton, Brzeski et al., 2017). Similarly, transient red wolves are likely driven to pairing quickly to acquire a territory and mate. Indeed, Hinton et al. (2016) stressed that red wolves and coyotes use the same habitats and, because transient wolves often bide in lower quality habitats proximate to wolf territories, they can destabilize coyote packs and displace coyotes from areas not occupied by resident wolves (but see Benson & Patterson, 2013). Consequently, individual red wolves compete with coyotes and other wolves for limited mates and space, and selection pressure on wolves and coyotes is likely greatest during the acquisition and defense of mates and territories. Because there are so few red wolves in the current population (Hinton, White et al., 2017), most wolves interact and compete with coyotes to acquire mates and defend territories, whereas historically wolves competed with other wolves for mates and space. In other words, when red wolves were more common, larger wolves likely had a selective advantage over smaller wolves when attempting to acquire and defend territories. Because coyotes greatly outnumber the reintroduced population, smaller red wolves currently have a selective advantage over larger wolves because small wolves are still large enough to outcompete coyotes for space, but are also capable of pairing with coyotes when wolf mates are not available. This is problematic for red wolf recovery because the ability of smaller red wolves, particularly females, to form congeneric pairs facilitates reproductive interference by coyotes (Gröning & Hochkirch, 2008; Mallet, 2005) and prevents wolf compensation of losses to mortality via reproduction (Hinton, White et al., 2017).

Patterns of assortative mating occur at the population level (Arnqvist et al., 1996; Crespi, 1989; Taborsky et al., 2009), and we suggest that assortative mating can be managed simultaneously with other population‐level processes (i.e., births, deaths, immigration, emigration) essential for population persistence. Specifically, factors influencing assortative mating also depend on population processes sensitive to anthropogenic mortality and small population sizes. For example, Brzeski et al. (2014) reported large inbreeding coefficients (average *f *=* *0.154) in wild red wolves and found a negative correlation between body size and inbreeding such that more inbred individuals were smaller. Inbreeding in the wild population is exacerbated by a small population size and high anthropogenic mortality, and those two factors are also correlated with hybridization (Bohling & Waits, 2015; Rutledge, White et al., 2012). Therefore, the USFWS may consider increasing abundance of red wolves in eastern North Carolina by focusing on mitigation of human‐caused mortalities (e.g., gunshot mortalities) and providing further protection of a core population of red wolves within the 5‐county Recovery Area, while also expanding recovery efforts beyond the Recovery Area to grow a large and robust regional wolf population. This approach could implement similar legal protection as those used in Ontario, Canada to protect eastern wolves (Benson et al., 2014; Rutledge, Patterson et al., 2010; Rutledge, White et al., 2012), which would increase red wolf abundance and improve pack structure while restoring selection pressures favoring larger‐sized red wolves to acquire and defend breeding territories from other wolves and not coyotes. Consequently, this would likely increase mean body sizes and home‐range sizes of wild red wolves and decrease hybridization rates with coyotes by reducing congeneric pairing.

## CONFLICT OF INTEREST

None declared.

## AUTHOR CONTRIBUTIONS

J.W.H. conceived the project, designed the experiment, organized and did field work, laboratory work, data analysis, and drafted the manuscript; J.L.G. contributed intellectually and edited/approved the manuscript; F.T.vM contributed to the project design, secured funding, and contributed intellectually and edited/approved the manuscript; M.J.C. contributed to the project design, secured funding, provided equipment, and contributed intellectually and edited/approved the manuscript.

## DATA ACCESSIBILITY

Data available from the Dryad Digital Repository: https://doi.org/10.5061/dryad.8rt51j0
